# Exercise Tolerance Can Be Enhanced through a Change in Work Rate within the Severe Intensity Domain: Work above Critical Power Is Not Constant

**DOI:** 10.1371/journal.pone.0138428

**Published:** 2015-09-25

**Authors:** Jeanne Dekerle, Kristopher Mendes de Souza, Ricardo Dantas de Lucas, Luiz Guilherme Antonacci Guglielmo, Camila Coelho Greco, Benedito Sérgio Denadai

**Affiliations:** 1 Centre for Sport and Exercise Science and Medicine, University of Brighton, Eastbourne, United Kingdom; 2 Physical Effort Laboratory, Sports Center, Federal University of Santa Catarina, Florianópolis, Brazil; 3 UNESP, Human Performance Laboratory, Rio Claro, Brazil; University of Rome Foro Italico, ITALY

## Abstract

**Introduction:**

The characterization of the hyperbolic power-time (*P*-*t*_*lim*_) relationship using a two-parameter model implies that exercise tolerance above the asymptote (Critical Power; CP), i.e. within the severe intensity domain, is determined by the curvature (*W’*) of the relationship.

**Purposes:**

The purposes of this study were (1) to test whether the amount of work above CP (*W*>CP) remains constant for varied work rate experiments of high volatility change and (2) to ascertain whether *W’* determines exercise tolerance within the severe intensity domain.

**Methods:**

Following estimation of CP (208 ± 19 W) and *W’* (21.4 ± 4.2 kJ), 14 male participants (age: 26 ± 3; *peak* $\dot{V}{\text{O}}_{2}$: 3708 ± 389 ml.min^-1^) performed two experimental trials where the work rate was initially set to exhaust 70% of *W’* in 3 (‘THREE’) or 10 minutes (‘TEN’) before being subsequently dropped to CP plus 10 W.

**Results:**

*W*>CP for TEN (104 ± 22% *W’*) and *W’* were not significantly different (*P*>0.05) but lower than *W*>CP for THREE (119 ± 17% *W’*, *P*<0.05). For both THREE (*r* = 0.71, *P*<0.01) and TEN (*r* = 0.64, *P*<0.01), a significant bivariate correlation was found between *W’* and *t*_*lim*_.

**Conclusion:**

*W*>CP and *t*_*lim*_ can be greater than predicted by the *P*-*t*_*lim*_ relationship when a decrement in the work rate of high-volatility is applied. Exercise tolerance can be enhanced through a change in work rate within the severe intensity domain. *W*>CP is not constant.

## Introduction

In cycle ergometry, the higher the power output (*P*), the shorter the time to task failure (*t*_*lim*_) so that the *P*-*t*_*lim*_ relationship is of a shape of a hyperbole within the severe intensity domain [1,2]. The performance of several exercises performed to task failure allows for an asymptote referred to as Critical Power (CP) and a curvature constant (*W’*) to be estimated using a two-parameter model (Eq 1) [2,3]. One interest in the application of the *P*-*t*_*lim*_ relationship would lie in its usefulness in predicting *t*_*lim*_ as long as the work rate requirement is greater than CP (‘*P*–CP’ in Eq 1 **)**, i.e. within the severe intensity domain [4]. Indeed, any prediction of *t*_*lim*_ from the modeling of the equivalent hyperbolic speed-time relationship in running [5,6] or *P*-*t*_*lim*_ relationship in cycle ergometry [3,7,8] is highly accurate. This relies on and supports the assumptions that while CP is rate-limited, *W’* is a fixed or capacity-limited amount of work [4,9–11]. Irrespective of the work rate requirement above CP, as long as *P* exceeds CP, *W’* is utilized at a rate determined by the difference between the required *P* and CP (‘*P* CP’ in Eq 1) so that at task failure, *W’* is fully utilized [4,6,11,12]. A strong determinant of *t*_*lim*_ in the severe intensity domain (i.e. when exercising above CP) should therefore be *W’* although this has never been directly evidenced.

$$t_{lim}\;=\;\frac{W'}{\left(P-CP\right)}$$ (1)

The most inquisitive design developed to test this hypothesis was that of Fukuba et al. [12] who manipulated the work rate during exercise performed above CP. Half of *W’* was to be expended in the first part of two varied work rate tests (117% and 134% of CP) before an increase (117% → 134%) or decrease (134% → 117%) to a new work rate. The latter was to be maintained till task failure. The total amount of work performed above CP (*W*>CP) during these two experimental conditions were not significantly different from *W’* as estimated from the modeling of the *P*-*t*_*lim*_ relationship leading the authors to conclude that “the work equivalent of *W'* is not affected by power output variations during exhausting cycle ergometry, at least in the *P* range of 100–134% of CP.”

Challenges to the work of Fukuba et al. [12] and the use of the *P*-*t*_*lim*_ relationship to predict performance arise when considering the literature on pacing strategies. Indeed, it has been shown that work production can be maximized, and exercise tolerance consequently enhanced, through work rate manipulation. This has been evidenced for positive strategies in particular [or fast-paced pacing [13]] and for performance lying within the severe intensity domain [14,15]. Some insights into the underlying mechanisms have been proposed: A faster $\dot{V}{\text{O}}_{2}$ kinetic response and consequently greater mean $\dot{V}{\text{O}}_{2}$ in the early part of a positive exercise, has been related to improvements in performance lasting from 2 to 5 minutes [14,15]. Such exercise would enhance the aerobic contribution to the total energy turnover. This aerobic contribution is represented by ‘CP x *t*_*lim’*_ in the linear equivalent of the hyperbolic *P-t*_*lim*_ model presented in Eq 1 (see Eq 2; *W*: total work done for a given *t*_*lim*_). Accumulated oxygen deficit (AOD) has been found unchanged in a positive *vs* constant-load strategy [15] suggesting that the anaerobic contribution to the overall work requirement (*W’* in the CP model) was unaffected by the pacing strategy. The different distribution in the utilization of AOD over the trials was evidenced in the work of Aisbett et al. [15] leading the authors to suggest that the development of fatigue often associated with the utilization of *W’* when considering the CP model [4], could be delayed in positive pacing strategies [15].

$$W\;=\;W^{'}+CP.\;t_{lim}$$ (2)

The present study aimed to challenge the application of the CP concept to varied work rate exercise performed within the severe intensity domain. The present experimental design is less ‘conservative’ than that of Fukuba et al. [12]. The experiment has been designed to test two different decrements in the work rate. In both conditions, a greater work rate was to be maintained in the first part of the performance trial (Part_1) [12]. This work rate was set to exhaust 70% of *W’* (as opposed to 50% in Fukuba et al., [13]) in the shortest (3 minutes; condition THREE) or longest possible times (10 minutes; condition TEN). Some pilot work evidenced the incapacity for some participants to maintain the work rate required to exhaust 70% of *W’* in less than 3 and more than 10 minutes (both extremes when working above CP [4]). The second part of the experimental trials was set at CP plus 10 W (Part_2) in order to target the lowest end of the severe intensity domain, while remaining confidently above CP (> upper limit of the 95% confidence interval). Based on the CP concept, one would hypothesize that both exercise would cease when the remaining 30% of *W’* is depleted so that the duration of Part_2 of the two performance trials do not differ significantly. Because for every second spent at CP plus 10 W, 30% of *W’* would be taxed by 10 J for each participant, thus irrespective of their CP or *W’*, it was further hypothesized that the greater the *W’* of the individuals, the greater the exercise tolerance (or *t*_*lim*_) as evidenced by a positive bivariate correlation between *W’* and the overall *t*_*lim*_.

## Methods

### Participants

Fourteen active men participating in cycling or triathlon volunteered to take part in this study (Age: 26 ± 3; weight: 76 ± 7 kg; *peak* $\dot{V}{\text{O}}_{2}$: 3708 ± 389 ml.min^-1^). All participants were briefed as to the benefits and risks of participation and gave their written informed consent to participate in the study, which was approved by the Tier 1 Ethics Committee of the University of Brighton, United Kingdom. All were familiarized with the laboratory testing procedures. Participants were instructed to arrive at the laboratory at the same time of day, in a rested and fully hydrated state, at least 3 h postprandial. They were also asked to refrain from caffeine and alcohol consumption 6 and 24 h before each test, and to avoid strenuous exercise in the 24 h preceding a test session. All were free of cardiac, metabolic or respiratory diseases.

### Equipment

The tests were performed on an electrically-braked cycle (Lode Excalibur Sport, Lode BC, Groningen, Netherlands). Seat and handlebar heights were kept constant over the sessions for each participant. The laboratory temperature was set at 20°C with 40–50% relative humidity. Heart rate was monitored every second using a telemetric heart rate monitor (Accurex +, Polar Electro Oy, Kempele, Finland). Pulmonary gas exchange was measured continuously using a breath-by-breath open-circuit system (Cosmed Quark PFTergo, Rome, Italy). Before each test, the O_2_ and CO_2_ analysis systems were calibrated using ambient air and a gas of known O_2_ and CO_2_ concentration according to the manufacturer’s instructions, while the gas analyzer turbine flowmeter was calibrated using a 3-l syringe. Respiratory gas exchange variables ($\dot{V}{\text{O}}_{2}$, $\dot{V}{\text{CO}}_{2}$, ${\dot{V}}_{\text{E}}$) were corrected to STPD and BTPS, displayed for every breath and then subsequently interpolated to provide one value per second. Blood samples were collected from the ear lobe into microcentrifuge tubes containing 50 μl NaF (1%) for the determination of capillary blood lactate concentration ([La]; YSI 2300 STAT, Yellow Springs, Ohio, EUA).

### Experimental design

The participants visited the laboratory for three stages of experimentation. Stage 1 involved the determination of lactate threshold (LT), peak oxygen uptake (*peak$\dot{V}{\text{O}}_{2}$*), and maximal power output (*P*_max_) followed by a familiarization to a constant-load test performed to task failure. Stage 2 consisted of the performance of four to five constant-load tests to task failure to determine CP and *W’*. Stage 3 consisted of two randomly assigned condition trials. All tests were separated by a minimum of 24 h. For all stages, pedaling frequency was kept at 90 ± 5 rpm. Participants were instructed to remain seated during each test. The study was completed within three weeks for all participants.

### Stage 1: Determination of LT, *peak* $\dot{V}{\text{O}}_{2}$ and *P*_*max*_

The initial power output was 60 to 100 W depending on the fitness level of the participant with an increase of 20 W every 3 minutes. The incremental test was stopped when the LT was surpassed or when [La] rose above 4 mmol.l^-1^. An examination of the [La]–power output relationship was used to determine LT. The highest work rate attained that was not associated with an elevation in [La] above baseline (resting) levels (less than 1 mmol.l^-1^), as determined by at least two observers, was designated as the work rate associated with LT [16].

After a rest period of 30 minutes, the participants performed a fast ramp test to exhaustion. The test began with an initial 5 minutes of cycling at 90% of their previously determined LT before the work rate increased by 5 W every 12 s (equating to 25 W.min^-1^), to the limit of tolerance. End Respiratory Exchange Ratio (RER) and heart rate were systematically above 1.10 and 90% of maximal predicted heart rate, respectively. The breath-by-breath data from each exercise test were filtered manually to remove outlying breaths, defined as breaths ± 3 SD from the adjacent five breaths. *P*_*max*_ and *peak* $\dot{V}{\text{O}}_{2}$ were defined as the highest averaged 15-s power output value, and the highest average 15-s $\dot{V}{\text{O}}_{2}$ value recorded during the incremental test, respectively. A familiarization to the constant-load test was performed following recovery from the incremental protocol.

### Stage 2: Determination of CP and *W’*

Participants performed a series of four constant-load tests to the limit of tolerance, each at different power outputs (from 75% to 105% of *P*_max_) chosen to elicit exhaustion in 3 to 15 minutes [1]. Each test was preceded by 5 minutes of warm-up at 90% of LT, 5 minute of passive rest, finally followed by 3 minutes of 20 W baseline pedaling. Participants were not informed of the imposed work rate, their performance times or heart rate. The only variable known to the subjects was their pedaling frequency. For each test, *t*_*lim*_ was taken as the elapsed time, in seconds, between the imposed exhaustive work rate and the time at which the participant could no longer increase their pedaling frequency back to the pre-set level after a fall by 5 rpm for more than 5 seconds for the second time during the test and despite strong verbal encouragement.

For each participant, the three equivalents of the 2-parameter model were used to fit the data and estimate CP and *W'* [17]. An iterative nonlinear regression procedure was used for the modeling of the hyperbolic *P*-*t*_*lim*_ relationship (Microcal Origin 7.5; Northampton, MA, USA). The CP and *W’* estimates from the three equations were compared to select the best fit using the model associated with the lowest standard error for *W’* (SE-*W’*). If required, a 5^th^ determination trial was performed at a different work rate and entered in the model to bring the SE of CP and *W’* below 2 and 10% of CP and *W’*, respectively. Data from the modeling is presented in Table 1. The work accumulated above CP (*W*>CP) was subsequently computed for each constant-load exercise.

**Table 1:** Characterization of the *P-t*_*lim*_ relationship.

Participant	Model	Number of tests	CP (W)	SE-CP (W)	*W’* (kJ)	SE-*W’* (kJ)
1	*P-t*_*lim*_^*-1*^	5	197	3.9	14.9	1.1
2	*P-t*_*lim*_	4	197	1.3	16.1	0.6
3	*P-t*_*lim*_	4	215	3.2	17.9	1.7
4	*P-t*_*lim*_	4	210	1.5	18.4	1.0
5	*P-t*_*lim*_	4	195	1.4	18.6	0.9
6	*P-t*_*lim*_	4	186	0.9	20.2	0.7
7	*P-t*_*lim*_	4	223	0.8	20.2	0.5
8	*P-t*_*lim*_	4	218	3.3	20.7	1.5
9	*W-t*_*lim*_	4	202	3.0	21.1	1.3
10	*P-t*_*lim*_	4	221	3.2	25.0	1.7
11	*P-t*_*lim*_	4	170	4.0	26.6	2.2
12	*P-t*_*lim*_^*-1*^	4	247	3.4	26.7	1.3
13	*P-t*_*lim*_^*-1*^	5	228	1.9	27.8	0.7
14	*P-t*_*lim*_	4	207	1.7	25.2	0.8
		**Mean**	**208**	**2.4**	**21.4**	**1.1**
		**SD**	**19**	**1.1**	**4.2**	**0.5**

### Stage 3: The two condition trials

Once CP and *W’* were determined, the work rates required for 70% of *W’* to be taxed in 3 minutes [CP + (0.70 x *W’)*/180] and 10 minutes [CP + (0.70 x *W’)*/600] were calculated. In the third stage of testing, participants had to maintain these work rates for the initial part of the test (Part_1), i.e. for either 3 minutes (condition THREE) or 10 minutes (condition TEN), before a change in the work rate to CP plus 10 W (Part_2). This new work rate was to be maintained to the limit of tolerance. Both tests were preceded by 5 minutes of warm-up at 90% of LT, 5 minutes of passive rest, finally followed by 3 minutes of 20 W baseline pedaling. For each test, *t*_*lim*_ was taken as the elapsed time, in seconds, between the imposed exhaustive work rate and the time at which the participant could no longer increase their pedaling frequency back to the pre-set level after a fall by 5 rpm for more than 5 seconds for the second time during the test and despite strong verbal encouragement. Blood samples were taken at rest and at the end of Part_1 and Part_2 for the measure of [La]. Part_1 was conducted twice to improve the signal-to-noise ratio in the $\dot{V}{\text{O}}_{2}$ response. Participants were informed of the test design prior to the commencement of the exhaustion trials and were therefore expecting the drop in the work rate between Part_1 and Part_2.

### Data analysis

The amount of work accumulated above CP (*W*>CP) was computed for both Part_1 (*W*>CP_(1)_) and Par_2 of each trial (*W*>CP_(2)_) and expressed in both kJ and % of *W’*. As for the incremental test, the breath-by-breath data from each exercise test were filtered manually to remove outlying breaths, defined as breaths ± 3 SD from the adjacent five breaths. The data for each individual was then interpolated (Microcal Origin 6.0, Northampton, MA, EUA) to provide 1 s values, and two data sets were time-aligned and averaged. The first 20 s of the data collection post the onset of exercise (i.e., the phase I response) was deleted, and a nonlinear least squares algorithm was used to fit the data thereafter [16]. A single-exponential model was chosen to characterize the $\dot{V}{\text{O}}_{2}$ responses dynamics following the onset of exercise [14,18], as described in Eq 3 where $\dot{V}{\text{O}}_{2}(t)$ represents the absolute $\dot{V}{\text{O}}_{2}$ at a given time *t; $\dot{V}{\text{O}}_{2}{}_{\left(\text{b}\right)}$* represents the mean $\dot{V}{\text{O}}_{2}$ at baseline; A_1_ the amplitude, TD the time delay, and τ the time constant of the $\dot{V}{\text{O}}_{2}$ response. The model fit was initially constrained to the first 60 s of exercise (i.e. 20–60 s). The window was then lengthened iteratively until the exponential model demonstrated a discernible departure from the measured response profiles (as judged from visual inspection of a plot of the residuals of the fit) [19,20]. In addition, a single-exponential model without a time delay and with a fitting window commencing at t = 0 s (equivalent to the mean response time—MRT) was used to characterize the kinetics of the overall $\dot{V}{\text{O}}_{2}$ response during the initial part of the two tests. AOD of the initial part of the test was calculated by subtracting the volume of O_2_ actually consumed from the predicted O_2_ volume for the total exercise time. The linear $\dot{V}{\text{O}}_{2}–P$ relationship from the lactate threshold test was used to calculate the latter [15].

$$\dot{V}O_{2}\left(t\right)\;=\;\dot{V}O_{2\left(b\right)}+A_{1}\times \left(1-e^{-t-TD/t}\right)$$ (3)

### Statistical analysis

Data are reported as mean ± SD unless stated otherwise. All statistical procedures were performed using SPSS (version 20.0, Chicago, USA) with the null hypothesis rejected at an alpha level of 0.05. The normal distribution (Kolmogorov-Smirnov test) was verified for each set of data. A two-way ANOVA with repeated measures was performed to identify condition (THREE and TEN) x time differences. A one-way ANOVA with repeated measures was computed to compare actual (THREE and TEN) and predicted values from the *P-t*_*lim*_ model. The compound symmetry, or sphericity, was checked using the Mauchly’s test. When the assumption of sphericity was not met, the significance of *F*-ratios was adjusted according to the Greenhouse–Geisser procedure. Significant differences were followed up using planned pair-wise comparisons employing the Bonferroni corrected post-hoc test. Relationships were explored using Pearson’s product-moment or partial correlations. Bland and Altman plots (1986) were used to determine the bias and limits of agreement between two sets of data when appropriate.

## Results

Work rate, *t*_*lim*_, and work accumulated above CP (*W*>CP) for both THREE and TEN are presented in Table 2. As expected, no significant difference was found between 70% of *W’* and the actual *W*>CP_(1)_ for THREE and TEN (*F* = 2.86, *P* = 0.11) with strong correlations, low bias and 95% limits of agreements between 70% of *W’* and *W*>CP_(1)_ (Table 2).

**Table 2:** Work rate, work done above CP and duration of both parts of the two experimental trials.

Condition	THREE			TEN		
mean ± SD	*Part_1*	*Part_2*	*Overall test*	*Part_1*	*Part_2*	*Overall test*
Work rate (W)	292 ± 28	219 ± 20	231 ± 19	234 ± 22*	219 ± 20	227 ± 18*
Work rate (% of CP)	140 ± 8	105 ± 1	111 ± 2	112 ± 2*	105 ± 1	109 ± 2*
Work rate (% of *P*_*max*_)	91 ± 3	68 ± 3	72 ± 3	73 ± 2*	68 ± 3	70 ± 2*
Actual total work done (kJ)	52.5 ± 5.0	219 ±97^$^	271 ± 101^$^	140 ± 13*	132 ± 65*	272 ± 76
Predicted total work done (kJ)	52.5 ± 5.2	126 ± 31	178 ± 36	140 ± 13	126 ± 31	266 ± 43*
Actual *W*>CP (kJ)	15.0 ± 2.7	10.6 ± 5.1^$^	25.6 ± 7.3^$^	15.2 ± 3.3	7.4 ± 5.8*	22.6 ± 8.4
Predicted *W*>CP (kJ)	15.0 ± 2.9	6.4 ± 1.3	21.4 ± 4.2	15.0 ± 2.9	6.4 ± 1.3	21.4 ± 4.2
*(Bias ± 95% limits of agreements (kJ))*	*(0*.*0* ± *0*.*6)*	*(4*.*2* ± *8*.*5)*	*(4*.*2* ± *8*.*5)*	*(0*.*5* ± *2*.*2)*	*(1*.*0* ± *9*.*8)*	*(1*.*5* ± *10*.*7)*
*[zero-order correlation coefficient (variance explained)]*	*[*.*99 (98%)]* ^*&&*^	*[*.*68 (46%)]* ^*&&*^	*[*.*83 (86%)]* ^*&&*^	*[*.*97 (94%)]* ^*&&*^	*[*.*66 (44%)]* ^*&&*^	*[*.*90 (81%)]* ^*&&*^
Actual *t*_*lim*_ (s)	180 ± 0	985 ± 393^$^	1165 ± 393^$^	600 ± 0	683 ± 487*	1283 ± 487
Predicted *t*_*lim*_ (s)		642 **±** 125	822 ± 125		642 **±** 125	1242 ± 125

* Significantly different to THREE (*P*<0.05);

^$^ Significantly different to predicted by the model (*P*<0.05);

^*&&*^ Significantly correlated (*P*<0.01)

**Fig 1:**
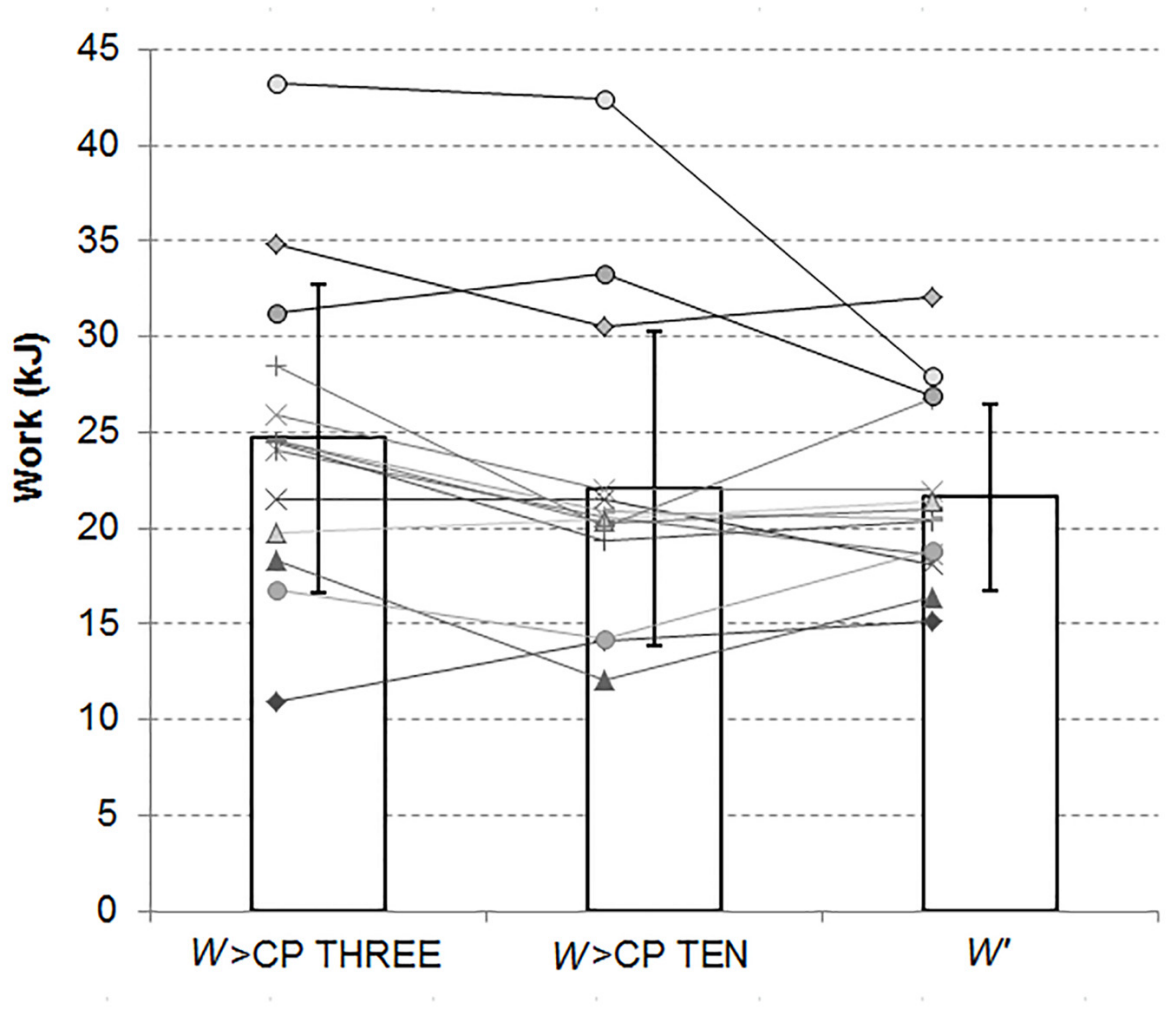
Mean ± SD alongside individual values for the two sets of *W*>CP and *W’*.

A significant difference was found between *W*>CP_(2)_ of THREE and TEN and 30% of *W’* (*F* = 7.77, *P*<0.01). *W*>CP_(2)_ for THREE was significantly greater than both *W*>CP_(2)_ for TEN (*P*<0.01) and 30% of *W’* (Table 2) with no significant difference between the latter two (*P* = 1). Significant bivariate correlations were obtained between the three sets of data (THREE *vs* TEN for *W*>CP_(2)_: *r* = 0.88, *P*<0.01; 30% of *W’ vs W*>CP_(2)_ for THREE: *r* = 0.68, *P*<0.01; 30% of *W’ vs W*>CP_(2)_ for TEN: *r* = 0.66, *P*<0.01). Bias ± 95% limits of agreement when comparing 30% of *W’* to *W*>CP_(2)_ were 64 ± 135% of 30% of *W’* for THREE and 15 ± 153% of 30% of *W’* for TEN (See Table 2 for absolute values). Fig 1 presents mean ± SD alongside individual values for *W*>CP accumulated during THREE and TEN, and *W’*. There was no significant difference found between *W*>CP for TEN and *W*>CP for the constant-load tests (*t* = 1.60, *P* = 0.11). The associated bias ± 95% limits of agreement were 0.4 ± 4.3 kJ or 2 ± 20% of *W’*. This bias was not significantly different to zero (*t* = 1.60, *P* = 0.11). Absolute bias ± 95% limits of agreement when comparing *W*>CP to *W’* for THREE and TEN are presented in Table 1. They were equal to 20 ± 40% of *W’* for THREE and 7 ± 50% of *W’* for TEN. The bias for TEN was not significantly different to zero (*t* = -1.0, *P* = 0.34) while a significant difference was found for THREE (*t* = -3.61, *P*<0.01).

Due to technical issues, the mono-exponential modeling of the response $\dot{V}{\text{O}}_{2}$ for Part_1 (Table 3) was only possible on 11 participants. Between-test differences were not significant for $\dot{V}{\text{O}}_{2}{}_{\left(\text{b}\right)}$ (*t* = 0.92, *P* = 0.19) but were significant for TD (*t* = 0.22, *P*<0.05), τ (*t* = 4.67, *P*<0.01), A_1_ (*t* = 3.65, *P*<0.01), and absolute $\dot{V}{\text{O}}_{2}$ (i.e., $\dot{V}{\text{O}}_{2}{}_{\left(\text{b}\right)}\;+\;{\text{A}}_{1}$) (*t* = 3.65, *P*<0.01). MRT was also significantly reduced (*t* = 2.92, *P*<0.01) while AOD was significantly smaller for THREE (*t* = 4.65, *P*<0.01). The ability for some participants to produce more work during Part_2 of THREE (i.e. ‘TEN-THREE’ difference in *W*>CP_(2)_) was not significantly related to the between-test difference in AOD (*r* = 0.06, *P* = 0.86) and the between-test difference in MRT (*r* = -0.51, *P* = 0.11).

**Table 3:** $\dot{V}{\text{O}}_{2}$ kinetics parameters for the first part of the two experimental trials.

Variables	THREE	TEN
$\dot{V}{\text{O}}_{2}{}_{\;(\text{b})}$ (l.min^-1^)	0.99 ± 0.11	0.97 ± 0.15
TD (s)	18.7 ± 3.3	15.5 ± 5.8*
τ (s)	22.8 ± 8.3	30.2 ± 8.2*
A_1_ (l.min^-1^)	2.35 ± 0.45	2.09 ± 0.26*
Absolute $\dot{V}{\text{O}}_{2}$ (l.min^-1^)	3.34 ± 0.48	3.07 ± 0.25
MRT (s)	55 ± 12	67 ± 13*
AOD (l)	2.41 ± 0.67	4.14 ± 1.56*

$\dot{V}{\text{O}}_{2}{}_{\left(\text{b}\right)}$—mean $\dot{V}{\text{O}}_{2}$ during the 60-s baseline period; TD—time delay; τ—time constant of $\dot{V}{\text{O}}_{2}$ kinetics (defined as the time required to attain 63% of the amplitude); A_1_ –amplitude of the $\dot{V}{\text{O}}_{2}$ response; Absolute $\dot{V}{\text{O}}_{2}$ or $\dot{V}{\text{O}}_{2}{}_{\left(\text{b}\right)}$ + A_1_; MRT—Mean Response Time, and; AOD—Accumulated Oxygen Deficit.

* Significantly different to THREE (*P*<0.05).

**Fig 2:**
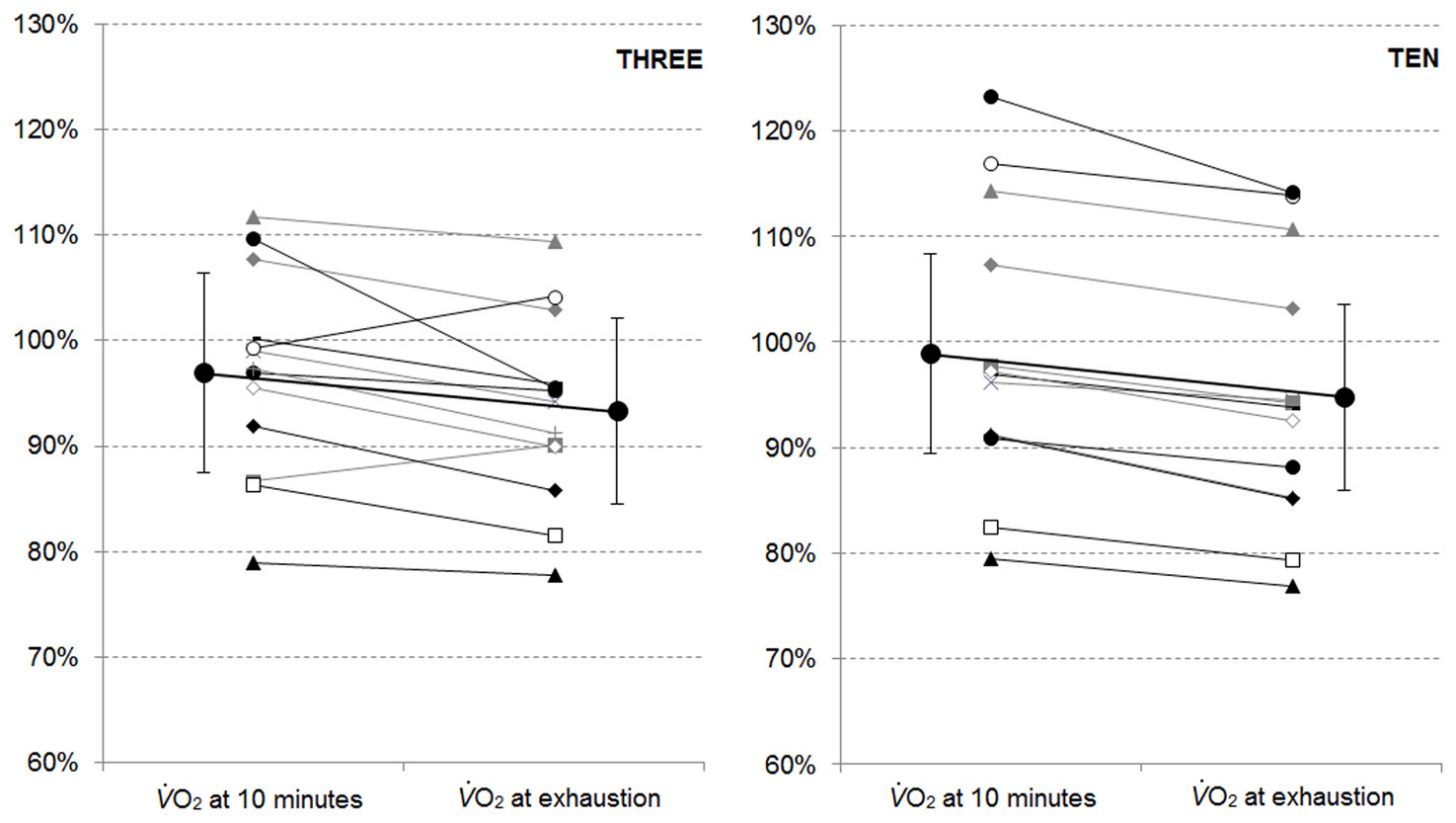
Mean $\dot{V}{\text{O}}_{2}$ expressed in % of *peak* $\dot{V}{\text{O}}_{2}$ recorded at the end of Part_1 and Part_2 of THREE and TEN. Group means are presented as open circles with standard deviation bars.

A two-way ANOVA with repeated measures revealed a time effect (*F* = 6.80; *P*<0.05), but no test effect (*F* = 0.35; *P* = 0.57) or interaction (*F* = 0.07; *P* = 0.80) for the [La] values reached at the end of Part_1 (THREE: 10.4 ± 2.7 mmol.l^-1^; TEN: 10.8 ± 2.0 mmol.l^-1^) and Part_2 of the two tests (THREE: 11.6 ± 2.7 mmol.l^-1^; TEN: 11.8 ± 2.3 mmol.l^-1^). The change in [La] throughout Part_2, per second of exercise, was not significantly different between the two tests (0.08 ± 0.16 mmol.l^-1^.s^-1^ *vs* 0.05 ± 0.07 mmol.l^-1^.s^-1^, *t* = 5.60, *P* = 0.59). Similarly, whether expressed in absolute or relative terms, the mean $\dot{V}{\text{O}}_{2}$ values recorded at the end of Part_1 (THREE: 95 ± 5%; TEN: 95 ± 7% of *peak* $\dot{V}{\text{O}}_{2}$) and Part_2 (THREE: 92 ± 5%; TEN: 93 ± 7% of *peak* $\dot{V}{\text{O}}_{2}$) were not significantly different between THREE and TEN (*F* = 0.05, *P* = 0.83) but changed over time (*F* = 15.2; *P*<0.01) with no interaction effect (*F* = 0.25; *P* = 0.63). For both experimental conditions, these mean $\dot{V}{\text{O}}_{2}$ values were not significantly different at the end of Part_1 (*P*>0.05) but were significantly lower than *peak* $\dot{V}{\text{O}}_{2}$ at the end of Part_2 (*P*<0.05). Individual trends are presented in Fig 2. Cycling efficiency or the $\dot{V}{\text{O}}_{2}$ / work rate ratio calculated at the end of Part_1 (THREE: 12.0 ± 0.8 ml.W^-1^.min^-1^; TEN: 15.0 ± 0.7 ml.W^-1^.min^-1^) and Part_2 (THREE: 15.5 ± 1.0 ml.W^-1^.min^-1^; TEN: 15.7 ± 1.0 ml.W^-1^.min^-1^), was significantly greater for TEN (*F* = 183, *P*<0.01), increased over time (*F* = 42.5, *P*<0.01) with a greater increase during Part_2 of THREE (*F* = 213, *P*<0.01).

Heart rate increased from the end of Part_1 to the end of Part_2 (THREE: 170 ± 13 and 177 ± 11 beats.min^-1^; TEN: 173 ± 14 and 176 ± 12 beats.min^-1^; F = 18.1; P<0.01) with no difference between the two tests (F = 1.92, P = 0.19) but a greater change depicted for THREE (F = 5.60, P<0.05). Minute ventilation increased over time (THREE: from 116 ± 14 to 122 ± 13 l.min^-1^; TEN: from 119 ± 19 to 123 ± 17 l.min^-1^; *F* = 6.40; *P*<0.05) but with no test difference (*F* = 0.62; *P* = 0.45) or interaction effect (*F* = 0.19; *P* = 0.67). This increase over time was the result of an increased breathing frequency (THREE: from 41.4 ± 12.5 to 51.5 ± 10.4 breaths.min^-1^; TEN: from 45.0 ± 12.9 to 51.5 ± 11.1 breaths. min^-1^; *F* = 22.7; *P*<0.01) despite a slight decrease in tidal volume (THREE: from 2.94 ± 0.63 to 2.41 ± 0.26 l.breath^-1^; TEN: from 2.76 ± 0.57 l.breath^-1^ to 2.44 ± 0.30 l.breath^-1^; *F* = 12.7; *P*<0.01). No test-difference was found for these two physiological variables (*P*<0.05) but an interaction was depicted for tidal volume (*F* = 13.7; *P*<0.01). The rates of change over time in these physiological variables were not related to the differences in work produced during THREE and TEN.

Zero-order and partial correlations between the two sets of *t*_*lim*_ and *W’* and CP are summarized in Table 4.

**Table 4:** Coefficient of correlation (variance explained) for the relationships between *t*_*lim*_, *W’* and CP.

	Correlation	*t*_*lim*_ for THREE	*t*_*lim*_ for TEN
*W’*	zero-order	0.71 (50%) **	0.64 (41) *
*W’*	Partial, controlling for CP	0.67 (45%)*	0.65 (42) *
CP	zero-order	0.44 (19%) ^n.s.^	0.75 (56%) **
CP	Partial, controlling for *W’*	0.33 (11%) ^n.s.^	0.76 (58%) **
*W’* and CP	Multiple (forced entry)	0.75 (55%) **	0.87 (75%) **

## Discussion

Exercise tolerance within the severe intensity domain can be enhanced through a variation of high volatility in the work rate. An initial exercise phase of much greater work rate, 3 minutes at around 140% before a drop to 105% of CP in the present study, increases the total amount of work (∼49%) predicted from the *P-t*_*lim*_ relationship. A more conservative work rate decrement with a smaller difference between the two phases of an exhaustive trial, yet still performed within the severe intensity domain (10 minutes at around 112% before a decrease to 105% of CP), leads to the performance predicted from the *P-t*_*lim*_ modeling. More work above CP than *W’* can be produced when a work rate decrement of high volatility is imposed (Fig 1) but this was not explained by a faster $\dot{V}{\text{O}}_{2}$ kinetic response in the present study. With *W’* and *t*_*lim*_ significantly correlated for both conditions (Table 4), the present study also demonstrates that *W’* can be a stronger determinant of these *t*_*lim*_ than CP (the case for THREE)._._ Interestingly, a large proportion of the variance in the *t*_*lim*_ for THREE remains undetermined.

*W*>CP was 70 ± 2% for Part_1 and 33 ± 21% of *W’* for Part_2 of TEN. The latter was not significantly different to the expected 30% of *W’* (Table 2 and Fig 1). These results for TEN are in accordance with the findings of Fukuba et al. [12] who also found exhaustive trials performed within the severe intensity domain to end when *W’* was fully utilized, even when the work rate was changing moderately during the exercise. Interestingly, the duration of our trial was much longer than those of Fukuba et al. [12] (∼ 20 *vs* ∼ 6 minutes). From an estimation of CP and *W’* using a 3-min all-out test [End Power (EP) and work done above EP (WEP) considered as surrogates for CP and *W’*, respectively], Chidnok et al. [11] also found the *P-t*_*lim*_ model to predict performance of ∼ 3 and 12 minutes accurately. WEP was not significantly different from predicted but with greater Coefficient of Variation (CV; 19–25%) than those reported for *t*_*lim*_ (3–8%). In agreement with these findings, in the present study, *W*>CP_(2)_ accumulated during TEN was not significantly different and correlated well with 30% of *W’* (Table 2) while the test lasted 1283 s on average, only 41 seconds longer than the prediction from the model (or 3.2% of *t*_*lim*_). Despite this low bias, the 95% limit of agreement was ± 487 s or one third of the mean *t*_*lim*._ CV computed for each individual led to rather large mean and standard deviation (20 ± 13%) and typical error was 24.7%. A prediction of long *t*_*lim*_ of varied-work rate within the severe intensity domain is therefore not as accurate as previously reported for constant-load tests of shorter duration [5,11]. This could be explained by the loss of accuracy in the prediction from the *P-t*_*lim*_ model as *t*_*lim*_ increases [5] as well as a possible impact of the change in work rate during TEN on the overall performance. The bias ± 95% limits of agreement between *W*>CP and *W’* was better for the constant load tests than for TEN, corroborating these findings (Table 2).

As for TEN, Part_1 of THREE was successful in exhausting 70% ± 2% of *W’*. However, *W*>CP_(2),_ and therefore overall *W*>CP, were greater than those predicted by the model (Table 2; +19% of *W’*; +4% only for TEN—the more conservative approach). For 12 of the 14 participants, *W*>CP was greater than the upper limit of the 95% CI associated with the estimation of *W’*. The participants in the present study could maintain Part_2 of THREE for ∼ 6 minutes longer than predicted (Table 2). This improvement in performance differs from the findings for TEN and previously reported [12], and may challenge the application of the CP concept to varied work rate exercise, but supports previous results showing that positive pacing strategies can improve exercise tolerance within the severe intensity domain [14,15,21]. Therefore, and in disagreement with the previous findings reported by Fukuba et al. [12], *W*>CP does not always hold constant when exercising above CP. In agreement, interventions such as moderate hypoxia [8], heavy-intensity priming exercise [18] or blood flow occlusion [22] have been shown to increase *W’* while CP was decreased [8,22] or unchanged [18]. These findings suggest that *W′* may be determined by other mechanisms than a finite amount of work, in consistency with emerging evidence [22–24]. Thus, *W′* may need to be better defined for the parameter to apply to physiological responses observed during varied work rate exercise.

**Fig 3:**
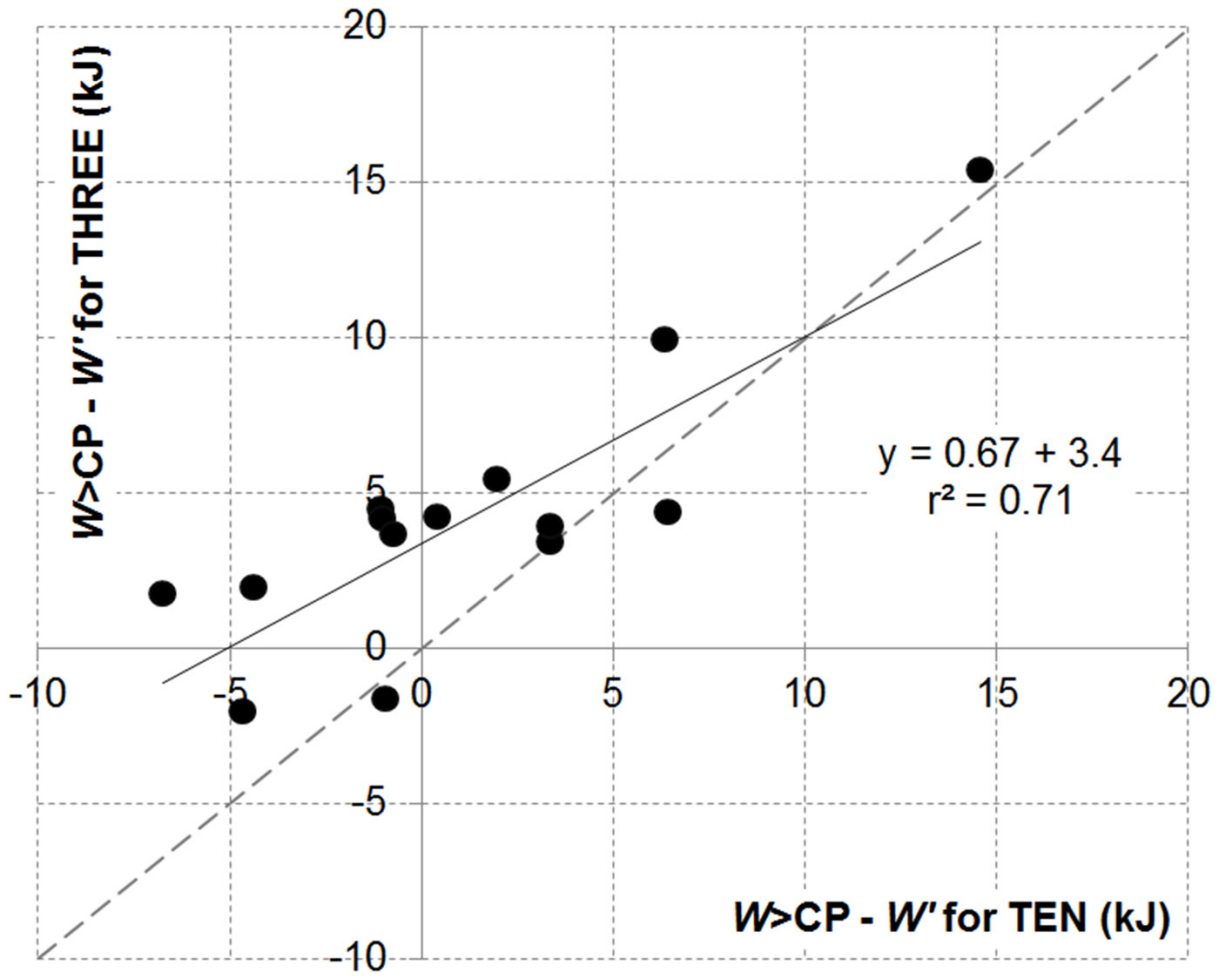
Scatter plots representing the differences between *W*>CP and *W’* for THREE against TEN. The dash line represents the line of identity.

The CP concept is based on a whole-body bioenergetic model [9] with key assumptions [2,4,25] that can be questioned [see [9] detailed review]. First, the anaerobic component of the overall energy supply (i.e. *W’*) is assumed constant. An estimation of ‘true’ anaerobic contribution to exercise is difficult [26] with current questionings of the reliability, accuracy and validity of AOD (see [27] for a review). With these limitations in mind, previous publications have reported no change in AOD [15,28,29] despite modifications in the rate of accumulation over a performance trial when the pacing strategy was manipulated [15]. Interestingly in the present study, the total volume of O_2_ consumed (61–64 l) and work produced were not significantly different between THREE and TEN leading to an O_2_ cost per Joule of ∼0.24 l.J^-1^ for both tests. This supports previous evidence for a similar anaerobic work capacity to be contributing to the overall work production, assuming cycling efficiency was kept constant. Secondly, the aerobic supply is assumed rate-limited in the 2-parameter CP model [2]. This assumption can be questioned as an increase in the aerobic energy supply has been put forward to explain the better performances recorded with a positive pacing strategy [14,15,29]: Greater volumes of O_2_ consumed and / or faster $\dot{V}{\text{O}}_{2}$ kinetic responses during the first part of fast-paced trials have been reported [10,14,15,21,30,31]. Part of the difference between *W*>CP and *W’* found in the present study for THREE could therefore, ‘in true’, be of aerobic nature. The required increase in CP to equal this difference would only need to be of ∼3.6 W (i.e. 4.2 kJ over 1165 s). The increase in CP (+15 W) by self-paced rather than the traditional fixed and pre-imposed work-rate exercise tests used to model the *P-t*_*lim*_ relationship supports this explanatory mechanism [7]. Unfortunately, this is not supported by the present study when analyzing changes in volumes of O_2_ or $\dot{V}{\text{O}}_{2}$ kinetic parameters. The third key assumption of the CP model is for cycling efficiency to be constant within the severe intensity domain, which remains unknown. None of our physiological measures explain the beneficial effect of the decrement in work rate during THREE. Of interest, differences in *W*>CP and *W’* were computed individually for both tests and a strong bivariate correlation was obtained (*r* = 0.83, *P*<0.01; Fig 3). Participants who performed better than expected for THREE also performed better than expected for TEN. This would need further exploration.

The previous paragraph discussed the present findings within the original Critical Power framework [9,25] the scientific community seems to be moving away from [22–24]. The physiological underpinning of *W’* has been particularly challenged over the past 10 years [24] with the notion of a fixed energy store [4,9,25] evolving toward a more ‘moldable’ work capacity. Exhaustion has been suggested to occur once accumulations of fatigue-related metabolites reach critical thresholds within the muscle cells as shown for H^+^ and P_i_ using ^31^P-MRS [11,32,33]. So any intervention delaying fatigue-related metabolites accumulations should enhance *W’*, and exercise tolerance as a consequence. In line with these new view on *W’*, one may speculate that one of the ‘priming’ effect of the first part of THREE is an increase in blood flow to type II fibers [24,34]. Oxygen delivery to the muscle cells could facilitate the aerobic energy turnover (and consequently increase CP) while a better muscular perfusion would also delay the cellular metabolic instability during the second part of the test [23]. The lack of significance in the $\dot{V}{\text{O}}_{2}$–related variables in the present study, partly because of a lesser efficiency at the end of part_1 of TEN (∼15 ml.min^-1^.W^-1^) when compared to THREE (∼12 ml.min^-1^.W^-1^) [23] could mask dissimilarities in the muscular vascular perfusion between the two experimental tests. In agreement with this assumption is the increase in *t*_*lim*_ when an imposed work rate lies within the lower part of the severe intensity domain, i.e. close to CP, similar to the work rate of the second part of our experimental trials (CP+10 W), following nitrate supplementation [35,36]. The nitrate supplementation resulted in a slight but not statistically significant increase in *W’* (+8.4%) and CP (+1.4%).

A more integrative approach to fatigue during exercise of severe intensity would also consider greater volume of oxygen ventilated (∼31 l for Part_1 of TEN *vs* 8 l for Part_1 of THREE) and possibly reactive oxygen species (ROS) produced during the first part of TEN [37]. More breaths taken (similar breathing frequencies and minute ventilation at the end of Part_1 of THREE and TEN while Part_1 was more than 3 times longer for TEN) would lead to greater respiratory muscle work [38,39]. More mechanical work produced (+ 67%) and consequently heat generated would demand an adequate but straining thermoregulatory response to keep the muscle cell cool [40]. The repetition of excitation-contraction coupling over time is also associated with extracellular and intracellular ionic disturbances (ROS, Na^+^, K^+^, Cl^-^, Ca^2+^) thought to contribute to peripheral fatigue [37,41]. So some elevated but similar metabolic and cardio-respiratory responses at the end of Part_1 of THREE and TEN could hide a more pronounced development of peripheral and overall fatigue after the first 10 minutes of TEN when compared to the initial 3 minutes of THREE, even though the same amount of *W’* was utilized (70%). Interestingly, heart rate at the end of the first part of THREE (∼168 beats.min^-1^) was slightly lower than that recorded at the end of the first part of TEN (∼173 beats.min^-1^), allowing for a greater increase during the second part of this test (+ around 7 vs 3 beats.min^-1^). The Critical Power framework may offer a reductionist approach for the rather complex integrative physiological responses underpinning exercise tolerance during whole body exercise [42].

The $\dot{V}{\text{O}}_{2}$ responses during both THREE and TEN demonstrate task failure can occur within the severe intensity domain without a systematic attainment of *peak* $\dot{V}{\text{O}}_{2}$ (Fig 2). One may argue the two tests were not performed till ‘true’ exhaustion with a voluntary decision made by each participant to end the test. It must be noted that these submaximal $\dot{V}{\text{O}}_{2}$ levels at the end of exercise were observed despite greater (THREE) or similar (TEN) *W*>CP than expected. Furthermore, the $\dot{V}{\text{O}}_{2}$ values at the end of the first part of both THREE and TEN were not different to *peak* $\dot{V}{\text{O}}_{2}$ but did decrease thereafter to reach submaximal levels at task failure (∼92–93%). These submaximal $\dot{V}{\text{O}}_{2}$ levels are also in line with previous reports [43–45], i.e. submaximal $\dot{V}{\text{O}}_{2}$ values as low as ∼88% of *peak* $\dot{V}{\text{O}}_{2}$ recorded at the end of exhaustive tests performed in the lower end of the severe intensity domain [43]. This challenges the physiological description of Critical Power as a threshold intensity above which exercise of sufficient duration will lead to attainment of a *peak* $\dot{V}{\text{O}}_{2}$ [46].

Three major determinants are traditionally offered to explain aerobic performance of long duration: *(1)* cycling efficiency, *(2) peak* $\dot{V}{\text{O}}_{2}$, and *(3)* the ability to maintain a high percentage of *peak* $\dot{V}{\text{O}}_{2}$ for a long time [or aerobic endurance, [47,48]. The theoretical framework of the CP concept offers for *W’* to govern the capacity for a higher percent of *peak* $\dot{V}{\text{O}}_{2}$ to be maintained for a long time when exercising within the severe intensity domain. Indeed, according to the CP model, exercise tolerance (i.e. *t*_*lim*_) is dictated by the size of *W’* for any given work rate above CP (*P*–CP; Eq 1), so that exercise ends when *W’* is fully utilized (as evidenced with TEN). The significant positive relationship between *W’* and the *t*_*lim*_ of both THREE and TEN supports this framework (Table 4). Furthermore, for two tests of similar durations (Table 2), *W’* becomes a better determinant of *t*_*lim*_ than CP for THREE although none of the two variables, even combined (55% of variance explained), explains well exercise tolerance for this condition. The variance in the *t*_*lim*_ for TEN is much better shared between *W’* and CP supporting for the 2-parameter modeling of the *P-t*_*lim*_ relationship to be challenged by a positive pacing strategy of high volatility. A negative relationship was reported by Billat et al. [49] between the running speed associated with *peak* $\dot{V}{\text{O}}_{2}$ and its associated *t*_*lim*_ (*r* = -0.36, *P*<0.05): The higher the speed, the shorter the associated *t*_*lim*_. The authors mainly discussed aerobic endurance while we hypothesized for the anaerobic capacity of the runners to potentially explain this result as well.

## Conclusion

Exercise tolerance can be enhanced by a positive pacing strategy in the severe intensity domain. The change in work rate has to be of high volatility with, in the present study, a decrease from 140% to 105% of CP at the third minute of exercise. This led to a ∼49% increase in the total amount of work when compared to those predicted from the hyperbolic *P-t*_*lim*_ relationship. The work accumulated above CP was greater than *W’* challenging the application of the CP concept to varied work rate exercise within the severe intensity domain. Although *W’* determines exercise tolerance better than CP in this domain of intensity, work accumulated above CP can be enhanced with a delay in the accumulation of fatigue-inducing metabolites as one proposed explanatory mechanism. A more mechanistic approach to the physiological mechanisms underlying the enhancement of exercise tolerance through a change in work rate within the severe intensity domain is required to investigate the present findings further.
